# Measuring antenatal counseling skill with a milestone-based assessment tool: a validation study

**DOI:** 10.1186/s12909-023-04282-5

**Published:** 2023-05-10

**Authors:** Michelle J. Bartlett, Rachel Umoren, Josephine H. Amory, Trang Huynh, Amanda J. H. Kim, Amanda K. Stiffler, Rossella Mastroianni, Ellie Ficco, Heather French, Megan Gray

**Affiliations:** 1 University of Washington School of Medicine, Seattle, 98105 USA; 2 Oregon Health & Science University, Portland, USA; 3 Children’s Hospital of Philadelphia, Philadelphia, USA

**Keywords:** Simulation, Antenatal counseling, Validity

## Abstract

**Background:**

Antenatal counseling for parents in the setting of expected preterm delivery is an important component of pediatric training. However, healthcare professionals receive a variable amount and quality of formal training. This study evaluated and discussed validity of a practical tool to assess antenatal counseling skills and provide evaluative feedback: the Antenatal Counseling Milestones Scale (ACoMS).

**Methods:**

Experts in antenatal counseling developed an anchored milestone-based tool to evaluate observable skills. Study participants with a range of antenatal counseling skills were recruited to participate in simulation of counseling sessions in person or via video with standardized patient actors presenting with preterm labor at 23 weeks’ gestation. Two faculty observers scored each session independently using the ACoMS. Participants completed an ACoMS self-assessment, demographic, and feedback survey. Validity was measured with weighted kappas for inter-rater agreement, Kruskal–Wallis and Dunn’s tests for milestone levels between degrees of expertise in counseling, and cronbach’s alpha for item consistency.

**Results:**

Forty-two participants completed observed counseling sessions. Of the 17 items included in the tool, 15 items were statistically significant with scores scaling with level of training. A majority of elements had fair-moderate agreement between raters, and there was high internal consistency amongst all items.

**Conclusion:**

This study demonstrates that the internal structure of the ACoMS rubric has greater than fair inter-rater reliability and high internal consistency amongst items. Content validity is supported by the scale’s ability to discern level of training. Application of the ACoMS to clinical encounters is needed to determine utility in clinical practice.

**Supplementary Information:**

The online version contains supplementary material available at 10.1186/s12909-023-04282-5.

## Introduction

Antenatal counseling for parents in the setting of expected preterm delivery is an important component of neonatal-perinatal medicine (NPM). The goal of these counseling sessions is to build trust with the expectant family, share key information, and have a collaborative discussion about goals of care for their baby [1]. There is a variable amount and quality of formal training in these critical communication skills during pediatric residency and NPM fellowship. Pediatric residents indicate that they have little experience with the communication skills for discussing prognosis and delivering difficult news and NPM fellows note significant gaps in their training surrounding communication and shared decision making with families [2–5]. Many learners disclosed that they had no formal education in communication skills during their training programs illustrating a deficit in necessary experience [6].

There are multiple methods available to teach antenatal counseling skills, including lecture-based didactics, online modules, observation of experienced faculty, and simulations with standardized patients; however, the former three are limited by learner engagement and faculty experience [4, 7, 8]. Simulations are an increasingly utilized tool for effective medical education and have been used successfully in the NPM community [9–13]. Surveys of NPM program directors showed that approximately 25–30% of programs use simulations with a majority reporting that simulations are overall effective as a tool for communication skills education [14, 15]. NPM fellows and nurse practitioners have similar sentiments about the importance of simulations as an integral element of their training programs [15, 16].

Antenatal consults require seamless integration of medical knowledge sharing with non-verbal and verbal communication skills [6, 17]. The Accreditation Council for Graduate Medical Education (ACGME) requires that NPM fellows demonstrate “competence in patient consultation;” however, there are no tools with reported validity evidence available to reliably evaluate the quality of antenatal counseling encounters [18]. We sought to develop and collect validity evidence of a practical system to evaluate antenatal counseling skills. We selected a milestone-based competency approach to mirror the ACGME’s competency-based medical education framework, emphasizing the learner’s ability to apply knowledge by performing specific, observable skills. Our tool was developed by integrating the skills needed to disclose difficult news, present “end-of-life” or palliative care options, and best practices for empathic communication with patients and effective communication with colleagues [1, 13, 18–20]. We also aim to present validity evidence as outlined by the Messick Framework including content, relationships to other variables, internal structure, response process, and consequences [21–23].

## Methods

### Competency tool development

The development of our tool started with a literature review of published best practices related to antenatal counseling, and an open discussion amongst our author group of expert physicians in neonatology, palliative care, and pediatrics to generate an initial list of domains important for the antenatal counseling encounter. VitalTalk™ is an evidenced-based communication training program used to teach skills in delivering “serious news” or discussing goals of care with a seriously ill patient. We utilized core Vital Talk™ skills to inform relevant domains [24, 25]. Our author group then deconstructed these domains into more specific elements and discussed how they could be assessed during counseling along a continuum of milestone anchors. We chose to use a global rating scale (GRS) format that provides examples of skills along a continuum rather than a yes/no checklist format to better be able to capture the complexities and nuances within an antenatal counseling environment [26–28]. Six domains were included in the ACoMS: 1) opening the visit, 2) setting the stage, 3) information sharing, 4) emotions and values, 5) communication, and 6) wrap up, with two to three individual elements within each domain (Additional file 1). Mirroring the ACGME milestone levels, the Antenatal Counseling Milestones Scale (ACoMS) anchors were: 1) novice, 2) advanced beginner, 3) competent, 4) proficient, and 5) expert [18].

As a second step to further develop and refine our tool, we collected anonymous survey data from 23 experienced counselors, our tool development panel (Table 2), who independently assigned milestone levels they felt best aligned with each of the elements within a given domain. These physicians were recruited from specialties that may perform a prenatal consult through email and verbal outreach based upon their willingness to participate in our project, in addition to their comfort and experience with the prenatal counseling encounter. General guidance was provided to the tool development panel to ensure they could connect the label to a typical learner level such that 1) novice = intern or resident with limited counseling background, 2) advanced beginner = resident or new fellow, 3) competent = senior fellow or less experienced attending, 4) proficient = experienced attending, and 5) expert = aspirational attending or attending who teaches counseling skills to others. Skill performance definitions were assigned to the milestone level closest to the mean and median of the tool development panel votes. In the case of a tie, the skill was allowed to encompass multiple milestone levels. These results were used to form the basis of the language within each element designating the milestone anchors for the tool.

### Study design

This study utilized a prospective cohort of physicians and physician assistant (PA) students who provided antenatal counseling during a patient simulation from July 2018 to October 2021. Pediatric residents, PA students, NPM fellows, and NPM attending physicians were eligible for the study. Participation was voluntary. Recruitment was performed through both verbal and email outreach during training sessions involving physicians from Seattle Children’s Hospital, the University of Washington School of Medicine, Oregon Health and Science University, the University of Hawaii, and British Columbia Children’s Hospital. Study size was based upon ability to recruit participants within the time frame of our study.

### Simulations

Prior to the simulations, participants were presented with a 20 min didactic introduction to the key elements and skills of an antenatal consult encounter [29]. This was initially in person and then transitioned to virtually during the COVID-19 pandemic. Two different individuals who also served as expert raters gave the didactic verbally with PowerPoint as a curriculum aid. These materials were created using existing resources and rater experience. Participants then performed an antenatal consult lasting up to 30 min with a simulated patient (SP). The simulated patients included volunteers experienced with acting as patients. All simulated patients received in-person or virtual training and written materials explaining their character, background, state of mind, and values prior to the encounter. They were instructed to have the same reaction through-out all encounters. The same case scenario of a first-time mother in preterm labor at 23 weeks with a singleton fetus was used for all simulations. No person involved in the case development was recruited as a participant. An experienced attending neonatologist on our author team observed each SP simulation either in-person or from a remote location using Zoom® (Zoom Video Communications Inc) software during the COVID-19 pandemic and provided an ACoMS rating for each element within each domain. Sessions were directly observed by two raters or video recorded for review by a second rater. Raters were experienced neonatologists who received an introduction to the tool and the same guidance as the tool development panel about connecting the label to a typical learner such that 1) novice = intern or resident with limited counseling background, 2) advanced beginner = resident or new fellow, 3) competent = senior fellow or less experienced attending, 4) proficient = experienced attending, and 5) expert = aspirational attending, attending who teaches counseling skills to others. During the study period, sessions were transitioned from in-person SP and participant counseling to virtual counseling to accommodate social distancing requirements due to the COVID-19 pandemic. Participants completed a survey that included demographic information, experience with prenatal encounters or communication training, and feedback related to the tool. Feedback was obtained about the clarity of the tool, the quality of self-reflection using the tool generated, and the utility of the tool for assessment of prenatal counseling skills.

### Participants

Validity evidence for the ACoMS was collected on a sample of providers including pediatric residents, physician assistant students, NPM fellows, and attending faculty in the neonatal intensive care unit (NICU).

### Statistical analysis

Rater and survey data was collected and stored by the REDCap electronic data capture tool hosted at the University of Washington. During tool development, the mean and median of the tool development panel votes for each skill performance definition for the ACoMS were calculated. Participant surveys and expert rater scores on the ACoMs milestone guide were analyzed using descriptive statistics. A weighted kappa test was utilized for inter-rater agreement between experienced counselors and a kruskal–wallis analysis (*p* < 0.05) was performed for group comparisons of milestone level scores. Post-hoc analysis was then performed using dunn’s test to determine if there were statistically significant differences between participant groups. Intraclass correlation (ICC) with cronbach’s alpha (α > 0.70) was used to evaluate inter-item correlation. Statistical analysis was done with Stata software release 17 (Stata Corp 2021).

## Results

There were 42 participants. The demographic and qualitative information of the participants is listed in Table 1. Over half of the participants had some previous experience with antenatal counseling, the most common being previous attendance of a lecture-style didactic. Only eight participants had ever received feedback on their antenatal counseling skills, and only two participants had the opportunity for self-evaluation after structured antenatal counseling encounters. Included in the *Posture/Room Set Up* element within the Starting the Visit domain was “preparing the room and sitting with the parents”, which was unable to be scored during video counseling simulations. The *Shared Decision Making* element in the Information Sharing domain was added after the first 11 participants based on feedback from faculty observers and participants that this was a key behavior not captured in the original tool. There is limited data for certain elements of our tool. For example, many raters left the *Summary* element unscored due to the element not being included by the participant. Fourteen sessions were not able to be scored by a second rater due to poor video quality.

**Table 1:** Demographics of participants

Role in the medical team (Total *N* = 42)	
Physician Assistant Student	2 (5%)
Resident	19 (45%)
Fellow	14 (33%)
Attending	7 (17%)
Post graduate years in training for trainees (median with IQR)	4 (3–4)
Years in practice for attendings (median with IQR)	7 (6–18)
Primary Field of Practice (*N* = 42)	
General Pediatrics	20 (48%)
Neonatology	21 (50%)
Palliative Care	1 (2%)
Previous training in critical conversations (*N* = 42)	
Lecture on counseling skills	23 (55%)
Counseling or communication workshop	16 (38%)
VitalTalk™ training	14 (33%)
VitalTalk™ faculty education	4 (10%)
Individual communication coaching	10 (24%)
Other relevant training	6 (14%)
Time spent in prenatal diagnosis clinic (*N* = 38)	
None	25 (60%)
1–2 weeks	7 (17%)
> 2 weeks	6 (14%)
Number of antenatal counseling experiences led in the past 6 months (median with IQR)	0.5 (0–5)

Demographic data of participants including previous experience with antenatal counseling and communication training

### Content validity

Based on Messick’s framework, content validity is best defined as how closely related the content of the tool is to the construct we are attempting to capture or measure [22]. Using the expertise of our author group, we identified six important domains of an antenatal counseling interaction (opening the visit, setting the stage, information sharing, emotions and values, communication, and wrap up) and the elements within each domain for a total of 17 individual elements to develop the ACoMS (Additional file 1) [1, 13, 18–20]. Our tool development panel included 23 counselors with a wide variety of antenatal counseling exposure, experience, and skills (Table 2). They voted on skill performance definitions, and we used the mean and median of their anonymous results to form the basis for our tool (Additional file 2). Skills with rewording suggestions from the panel were also reviewed and reworded for clarity, consistency, and efficiency by the author group. The preliminary milestones elements were piloted with 11 participants. The author team reviewed the pilot sessions, expert observer feedback, and participant input and added 2 additional skills elements to the ACoMS that were consistently called out as missing from the results of the content expert panel (Additional file 2).

**Table 2:** Demographics of the tool development panel

Field^a^ (Total *N* = 23)	
Neonatology	21 (91%)
Palliative Care	1 (4%)
Maternal Fetal Medicine	2 (8%)
Level of training	
Attending	19 (83%)
Fellow	4 (17%)
Years in respective subspecialty (median with IQR)	8 (4–33)
Formal role in Prenatal Clinic	4 (17%)
Self-identified gender	
Female	11 (48%)
Male	12 (52%)
Racial Identity^a^	
Asian	2 (9%)
White	21 (91%)
Ethnicity	
Hispanic or Latino	1 (4%)
Types of counseling routinely performed^a^	
Peri-viability	19 (83%)
Congenital conditions expected to require surgery	15 (65%)
Congenital conditions expected to require medical care	17 (74%)
Prenatal genetic testing	14 (61%)
Other	4 (17%)
Counseling settings^a^	
Inpatient	21 (91%)
Outpatient	13 (56%)
Previous counseling training^a^	
Prenatal counseling workshop	4 (17%)
Communication training (eg. Vital Talk)	16 (70%)
Communication educator training	8 (35%)
Prenatal counseling simulations	10 (43%)
Observed expert counselors	22 (96%)
Individual coaching on counseling/communication	9 (39%)
Other	2 (9%)
Prenatal counseling sessions performed in the past 6 months (median with IQR)	15 (9–26)

Demographic data of the Tool Development Panel including experiences with antenatal counseling in a variety of settings and counseling training

^a^Fields not mutually exclusive

### Relationship to other variables

**Fig. 1:**
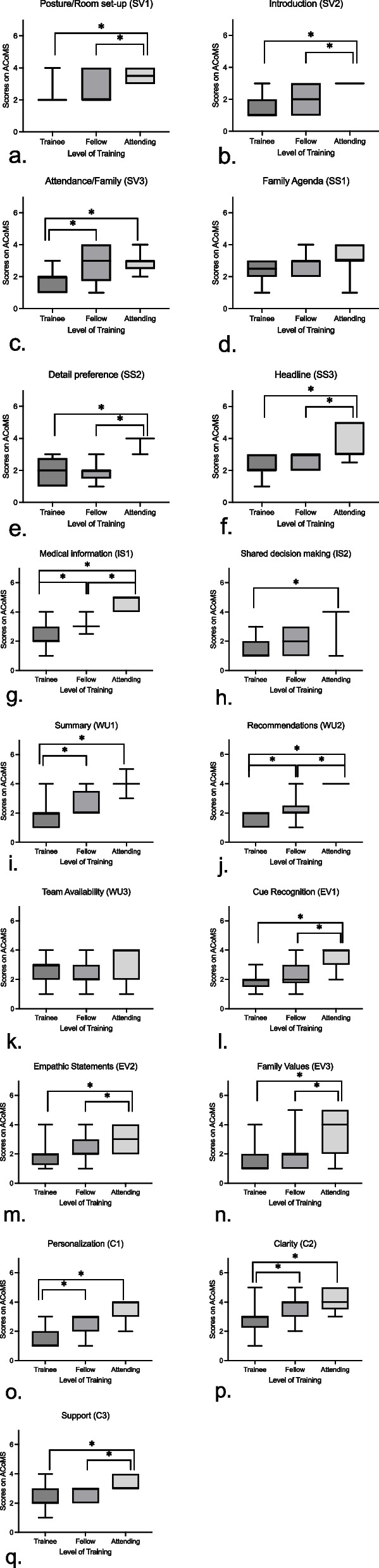
Analysis of ACoMS by level of training

Relationship to other variables attempts to identify whether the measurements on the tool correlate with the underlying construct we are attempting to evaluate; this roughly aligns with the framework of construct validity proposed by Cronbach and Meehl [22, 30]. Fifteen out of 17 items met statistical significance for a difference between level of training as a group (Table 3). All of the elements with statistically significant *p*-values from the Kruskal–Wallis have statistically significant differences between trainees and attendings scores on the ACoMS (Fig. 1). Most elements also demonstrated statistically significant differences in scores in at least one of the following group pairings: trainees vs. fellows or fellows vs. attendings (Fig. 1). The two elements (domains) that that did not meet statistical significance included: *Family Agenda (*Setting the Stage*)* and *Team Availability* (Wrap Up).

**Table 3:** Validity evidence

**Domains**	**Elements (code)**	**Rater Observations (N)**	***P*** value*	**Level of agreement (weighted κ)**	**Cronbach’s Alpha (α > 0.70)**
**Starting the Visit**	*Posture/Room set-up (SV1)*	32	**0.001**	**Fair (0.30)**	0.92
	*Introduction (SV2)*	42	**0.000**	**Fair (0.35)**	0.92
	*Attendance/Family (SV3)*	42	**0.002**	**Moderate (0.57)**	0.93
**Setting the Stage**	*Family Agenda (SS1)*	41	0.104	**Fair (0.38)**	0.93
	*Detail preference (SS2)*	41	**0.000**	**Moderate (0.45)**	0.92
	*Headline (SS3)*	41	**0.001**	**Fair (0.28)**	0.92
**Information Sharing**	*Medical information (IS1)*	42	**0.000**	**Moderate (0.52)**	0.92
	*Shared decision making (IS2)*	15	**0.049**	Slight (0.14)	0.92
**Wrap Up**	*Summary (WU1)*	30	**0.005**	Slight (0.15)	0.92
	*Recommendations (WU2)*	41	**0.000**	**Moderate (0.43)**	0.92
	*Team Availability (WU3)*	41	0.087	**Fair (0.28)**	0.93
**Emotions and Values**	*Cue Recognition (EV1)*	41	**0.002**	**Moderate (0.45)**	0.92
	*Empathic Statements (EV2)*	42	**0.013**	Slight (0.15)	0.92
	*Family Values (EV3)*	42	**0.012**	**Moderate (0.45)**	0.92
**Communication**	*Personalization (C1)*	42	**0.000**	**Moderate (0.58)**	0.92
	*Clarity (C2)*	42	**0.003**	Slight (0.20)	0.93
	*Support (C3)*	42	**0.023**	Slight (0.12)	0.93

Validity evidence as demonstrated by item scaling amongst skill level (kruskal wallis *p*-value), interrater reliability (weighted κ), and internal consistency (cronbach’s α). Bolded values indicate statistically significant results

^*^Kruskal Wallis *p* value (*p* < 0.05)

Post-hoc analysis of each element with dunn’s test to determine differences amongst training level of participants for each element demonstrated with box and whisker plots using the mean, IQR, minimum, and maximum. The shorthand code for each element is placed within parenthesis as related to Table 3 and Additional file 2. The brackets with a * denote a statistically significant difference (alpha = 0.05; Reject hypothesis if p = alpha/2) between levels of training using dunn’s test. Non statistically significant findings were not added to the graphs. We did not include statistically significant dunn test notations if the kruskal–wallis *p*-value was not significant. The “trainee” group includes both pediatric residents and physician assistant students.

### Internal structure

Internal structure is best defined as the consistency and relationship among data items which is similar to the framework for reliability [22, 23]. The ICC using cronbach’s alpha for inter-item consistency was significant for all items (Table 3). Of the weighted kappas evaluating interrater reliability for the 17 items, 5 had slight agreement (0.01–0.20), 5 had fair agreement (0.21–0.40), and 7 had moderate agreement (0.41–0.60) (Table 3). Two of the elements (*Shared Decision Making* and *Summary*) with slight agreement were limited by a smaller number of data points (15 and 30, respectively).

### Response process

Response process is best defined as whether the participant’s performance or understanding is consistent with the construct as defined by the investigators [22]. Of the participants who completed the feedback portion, all indicated that the tool was clinically useful. Almost all (97%) participants stated that the tool is clear. More than 80% of participants rated the tool as “very good” or “excellent” for overall assessment of prenatal counseling skills.

### Consequences

Consequences are best defined as the intended or unintended effect of the assessment [22, 23]. Our tool did not have a pass or fail denomination and no percentage, or numerical scores were utilized. Individual areas with more novice performance were shared in the context of areas for further development, rather than poor performance or failure. To encourage positive feedback, the tool offers areas for the rate to identify specific examples that a participant performed well within each element in addition to noting their biggest strength at the bottom of the tool (Additional file 1). Feedback generated from raters’ use of the tool was overall positive. Of the participants who completed the feedback portion, all indicated that the quality of self-reflection from the tool was good.

## Discussion

Using Messick’s validity framework we have presented validity evidence for the results of the novel ACoMS in a simulated antenatal counseling encounter within “five sources” including content validity, internal structure, relationships with other variables, response process, and consequences [21–23]. The majority of tool elements including *Posture/Room Set-up, Introduction, Attendance/Family, Detail Preference, Headline, Medical Information, Recommendations, Cue Recognition, Family Values, and Personalization* demonstrated ability to distinguish level of training, reliability across raters, and inter-item consistency. Participants across a variety of levels of training found the tool useful, clear, and were pleased with the insight it generated during feedback after simulated prenatal counseling sessions. Consistent with the literature, this supports the idea that simulations are valuable educational tools for building communication skills, and this tool will allow educators to fill an identified gap in communication training for their pediatric residents, physician assistants, fellows, and faculty [13–16]. The ACoMS is unique in that no previous GRS for evaluating antenatal counseling encounters has been published with established validity evidence within a modern framework. Previously reported tools published in the literature were checklists to evaluate medical information and communication strategies during antenatal counseling encounters; however, none included a GRS [10, 31]. Additionally, the lack of tools or measures in the literature with validity evidence to evaluate antenatal counseling skills limits the ability to measure the quality and impact of educational programs directed at teaching these challenging skills.

The methodical and evidence-based development of this tool using a content expert panel separate from our author group provides evidence of content validity. Most elements demonstrated that scoring higher on the tool discerns level of training, which is what we would expect on a GRS tool scoring along a continuum from novice to expert (Fig. 1). The weighted kappa demonstrates overall reliability amongst reviewers with two-thirds of the items having fair or greater agreement. ICC was significant for all elements demonstrating consistency amongst the elements. Our tool was overall rated as useful and easy to understand by participants. Our tool is not intended to be used as a metric for advancement or competency; rather its purpose is educational in a low stakes, “safe” environment evaluating performance during simulation case scenarios. Further, our tool provides unique and individualized feedback about areas of improvement in a simulated antenatal counseling environment. The simulation environment also allows for protection of vulnerable patient populations and the opportunity to provide a variety of situations with differing variables (E.g. religious beliefs, racial identity, ethnicity, gender identity, and preferred language) [13].

### Limitations

One limitation of this tool surrounds the ability to establish reliability with only 7 elements having moderate level of agreement. There are a number of encounters without scores from two experienced raters and some items which were not scored at all in certain encounters, which limits the number of completed data sets to be analyzed and affected reliability measurements. With a larger sample size with complete scoring data from two raters, there may be an increase in the level of agreement of those other elements. There is always a risk of conscious or unconscious bias concerning the subjectivity of raters, particularly as raters were not blinded to participants’ identities. Also, our tool has between three to five choices for each element to be rated upon with some elements having scale anchors that span multiple milestone levels due to ties from our tool development panel (Additional file 1). This results in a lack of consistency in scoring choices for each element, which makes comparing data metrics difficult, including reliability.

In addition, approximately halfway through our study period, we had to switch from in-person encounters to encounters via the Zoom platform, which could have introduced bias in the scoring of our raters. Performance by the individual was limited for certain elements over the Zoom platform compared to in-person encounters. For example, within the Setting the Stage domain, one’s posture and position within the room (ie. standing vs. sitting, location of other team members or patient’s family/friends) is an important element which is limited on the online platform. It may also have limited the scores within the Emotions and Values domain due to the difficulty of recognizing non-verbal cues online or inability to respond as one might during an in-person encounter, such as providing tissues or a gentle touch. However, as COVID precautions are lifted and simulations may return to in-person activities, this may no longer be an issue.

In addition, we added elements after initial testing which impacts our ability to interpret our data and compare scores, particularly for the Information Sharing domain. Replication of this study could be resource intensive, considering the cost and time to train both consistent raters and simulated patients. As this is the first proposed validity evidence data for antenatal counseling, we are unable to compare our scoring results to any field “gold standards”; thus, surrogate measures, such as level of training, were used in our analysis.

### Conclusion and next steps

In conclusion, we use Messick’s framework to present validity evidence as demonstrated by our tool being grounded in literature review, expert opinion, and institutional standards (content validity), most items having fair or greater reliability amongst reviews (internal structure), most items scaling with level of training (relationship to other processes), the clarity of the tool (response process), and the ability to provide feedback without a pass/fail system (consequences) [21].

Future studies of the tool during real-patient encounters are planned to assess how these skills will translate to clinical practice. Use of the tool with different scenarios featuring preterm and term infants at different gestational ages, with and without congenital anomalies, is anticipated to establish validity across a wide range of clinical encounters. In addition, we think that this tool could be utilized and integrated into training with our interprofessional colleagues including neonatal nurse practitioners who also may be involved with the prenatal consult. While some elements had poor interrater reliability and inability to consistently scale by level of training, our author group believes that *Shared Decision Making, Summary, Family Agenda, Empathic Statements*, *Clarity*, and *Support* are integral to the antenatal counseling encounter and should continue to be included in the tool pending further collection of validity evidence and a larger sample size. Our author group also hopes to utilize this tool in simulations using the Virtual Antenatal Encounter and Standardized Simulation Assessment (VANESSA), a screen based virtual standardized patient who is capable of showing emotions through animation and reacting to a variety of emotional responses [32]. Our tool is potentially useful to NPM training programs and could be extended to use in general pediatrics, obstetrics, maternal–fetal medicine, palliative care, genetics, and other specialties who meet with parents expecting preterm or ill infants. This tool has utility for individual evaluation, identification of individuals who may need more guidance, and use as a benchmark for whether participants are meeting their educational goals.

## Supplementary Information

**Additional file 1.** The Antenatal Counseling Milestones Scale (ACoMS). This file is a copy of the final tool used in our study to evaluate participants during their simulated patient sessions of an antenatal counseling encounter. There are 6 domains with 17 total elements. In addition, there is column on the righthand side for raters to notate specific examples they have observed during the encounter. There is also room at the bottom of the tool for further comments specifically related non-verbal communication skills and identifying the participants biggest strength.

**Additional file 2.** Content expert panel results on the milestone-based anchors for each element. This file shows the mean and medianresults of the content expert panel and the resulting milestone outcome, or element which can be correlated to the codes as seen in Table 3 or Fig. 1.

## Data Availability

The datasets used and analyzed during the current study are available from the corresponding author on reasonable request. Data is required to remain with the study team by IRB request.

## Abbreviations

ACoMS: Antenatal Counseling Milestones Scale
NPM: Neonatal-Perinatal Medicine
ACGME: Accreditation Council for Graduate Medical Education
GRS: Global rating scale
PA: Physician’s assistant
SP: Simulated patient
NICU: Neonatal intensive care unit
ICC: Intraclass correlation
VANESSA: Virtual Antenatal Encounter and Standardized Simulation Assessment
