# Supplementary material for: Measuring antenatal counseling skill with a milestone-based assessment tool: a validation study
Source: BMC Med Educ. 2023 May 10;23:325. doi: 10.1186/s12909-023-04282-5 (PMC10170031; doi:10.1186/s12909-023-04282-5)
Supplement: Supplementary file 1 — Additional file 1. The Antenatal Counseling Milestones Scale (ACoMS). This file is a copy of the final tool used in our study to evaluate participants during their simulated patient sessions of an antenatal counseling encounter. There are 6 domains with 17 total elements. In addition, there is column on the righthand side for raters to notate specific examples they have observed during the encounter. There is also room at the bottom of the tool for further comments specifically related non-verbal communication skills and identifying the participants biggest strength. [file 12909_2023_4282_MOESM1_ESM.pdf]

Novice	Advanced Beginner	Competent	Proficient	Expert	
**Starting the visit**					**Examples**
Stands for discussion	Prepares by sitting and identifying extra seats if needed	Eliminates distractions, brings tissues if appropriate	Reorganizes room/providers/family seating to facilitate discussion, ensures everyone is comfortable		
Introduces self by name to patient and explains one's own role	Introduces team and explains roles of team members and self[1]	Builds rapport and trust through introduction and explanation of role in care team			
Focuses family introduction on 1 person, omits baby's name	Asks to be introduced to family present, asks for baby's name	Asks to be introduced to everyone, seeks family preferences for who should be present, offers phone in option when appropriate, asks baby's name, permission to use it, and uses name in discussion			
**Setting the Stage**					
Focuses on personal agenda without assessment of family goals or understanding	Presents personal goals and agenda for conversation prior to a basic assessment of family understanding	Assesses family goals, agenda/questions, and understanding before giving medical information	Assesses and integrates family goals into a shared agenda, elicits family's understanding of the situation and context		
Family preferences for level of detail not identified	Family preferences identified only during the discussion	Family preferences identified at the start, applied throughout	Assess family preferences on level of detail (big picture or more detailed) and uses this to guide the discussion		
Uses indirect approach, avoids stating the headline	Presents medical information, but unclear headline	Delivers clear headline without jargon	Delivers clear headline without jargon and acknowledges ambiguity	Delivers clear, jargon free headline tailored to family context and acknowledges ambiguity	
**Information Sharing**					
Overlooks or is unclear with critical medical information, provides overly detailed medical information	Includes but does not highlight most critical piece of medical information, relies heavily on detailed descriptions of medical problems	Highlights most critical piece of medical information, uses more detailed descriptions of medical problems than requested by family	Focuses on the most critical pieces of medical information, occasionally uses more details than requested by family	Focuses on the most critical pieces of medical information, tailors level of medical details to family's preferences and adjusts as needed	
Provides limited or unclear information about options, decisions for care	Provides clear information about options/decisions	Introduces options/decisions with shared decision making	Uses knowledge of family values to guide shared decision making, navigates family uncertainty or difficulty with decisions		
**Wrap Up**					
Provides minimal summary of the discussion and assessment of family understanding	Provides a basic summary	Provides a basic summary, including next steps and asks about understanding	Reiterates headline, key decisions, and next steps in summary, confirms family understanding	Uses the summary to highlight key information, decisions, and next steps, ensures family agenda was met, confirms understanding	
Makes or withholds recommendations inappropriately	Specific recommendations made only if requested/appropriate		Specific recommendations are made based on patient/family's preferences and values only if requested/appropriate		
Omits explaining team availability	Explains team availability	Explains team availability with reassurance	Explains team availability with reassurance and shares how to access team for follow up questions		

Novice	Advanced Beginner	Competent	Proficient	Expert	
**Emotions and Values**					**Examples**
Acknowledges few emotional cues and continues with medical information	Recognizes obvious emotion cues and responds	Recognizes subtle emotional cues and responds	Notices when patient disengages, withdraws or freezes and responds effectively to re-engage them, recognizes emotional cues buried in questions or statements and responds		
Uses few empathic statements	Uses occasional empathic statements at times of high emotion	Predominantly uses empathic statements at times of high emotions and occasionally throughout the rest of the discussion	Uses empathic statements throughout the discussion and layers information delivery with responses to patient's emotion to guide patient through discussion, built rapport, and gather data about the patient/families needs[2]		
No discussion of values, focuses instead on options	Asks at least one specific question about values	Elicits multiple, specific values	Asks open ended questions and then clarifying questions to fully illuminate the values	Elicits multiple values and aligns with values to create family centered plan	
**Communication**					
Assumes patient understanding	Checks once for understanding, reiterates information around that point	Checks for understanding multiples times including at the end of the end of the conversation to ensure key points were communicated	Personalizes the discussion by identifying and adjusting to family understanding throughout the conversation and modifies progress of discussion based on results<br><br>Uses terminology of family to guide the conversation adapting information to provide counseling in a highly personalized way		
Uses medical jargon extensively, responds to requests for clarification with non-colloquial words	Uses medical jargon extensively, responds to requests for clarification with less jargon	Uses occasional jargon and responds to requests for clarification without jargon, notices signs of not understanding	Minimal use of medical jargon and explains any non-colloquial words or acronyms without prompting	Very minimal medical jargon and explains all non-colloquial words and acronyms without prompting	
Offers minimal supportive statements	Offers occasional supportive statements during times of high emotion	Offers supportive statements throughout discussion	Frequently offers supportive statements and reiterates support during summary at the end of the discussion, offers additional supportive services (SW, pastoral care, etc) as needed		

Other comments:

Use of silence?
Body language?
Eye contact?
Use of touch?

Biggest strength:
