# Supplementary material for: Measuring antenatal counseling skill with a milestone-based assessment tool: a validation study
Source: BMC Med Educ. 2023 May 10;23:325. doi: 10.1186/s12909-023-04282-5 (PMC10170031; doi:10.1186/s12909-023-04282-5)
Supplement: Supplementary file 2 — Additional file 2. Content expert panel results on the milestone-based anchors for each element. This file shows the mean and medianresults of the content expert panel and the resulting milestone outcome, or element which can be correlated to the codes as seen in Table 3 or Fig. 1. [file 12909_2023_4282_MOESM2_ESM.docx]

**Additional File 2. Content expert panel results on the milestone-based anchors for each element.**

| **Sub-Group** | **Skill** | **Mean Level ± SD** | **Median Level (IQR)** | **Milestone Outcome** | **Skill** | **Mean Level ± SD** | **Median Level (IQR)** | **Milestone Outcome** |
| --- | --- | --- | --- | --- | --- | --- | --- | --- |
| **Starting the Visit** | SV1-a | 1 ± 0.0 | 1 (1,1) | **SV1-1** | SV2-c | 2.1 ± 0.8 | 2 (1,3) | **SV2-2** |
|  | SV1-b | 1.9 ± 0.6 | 2 (1.5,2) | **SV1-2** | SV2-d | 2.9 ± 0.9 | 3 (2,4) | **SV2-3** |
|  | SV1-c | 2.8 ± 1.0 | 3 (2,3.5) | **SV1-3** | SV3-a | 1.1 ± 0.5 | 1 (1,1) | **SV3-1** |
|  | SV1-d | 3.5 ± 1.0 | 4 (3,4) | **SV1-4** | SV3-b | 1.9 ± 0.9 | 2 (1,2) | **SV3-2** |
|  | SV2-a | 1.1 ± 0.5 | 1 (1,1) | **SV2-1*** | SV3-c | 2.3 ± 0.9 | 3 (1.5,3) | **SV3-3*** |
|  | SV2-b | 1.5 ± 0.7 | 1 (1,2) |  | SV3-d | 2.9 ± 1.2 | 3 (1.5,4) |  |
| **Setting the stage** | SS1-a | 1.1 ± 0.2 | 1 (1,1) | **SS1-1** | SS2-c | 3.0 ± 0.8 | 3 (2.5, 4) | **SS2-3** |
|  | SS1-b | 1.9 ± 0.7 | 2 (1,2) | **SS1-2** | SS2-d | 3.8 ± 0.7 | 4 (3,4) | **SS2-4** |
|  | SS1-c | 2.9 ± 0.8 | 3 (2.5,3) | **SS1-3** | SS2-e | 4.2 ± 0.6 | 4 (4,5) | **Com1-4*^** |
|  | SS1-d | 3.6 ± 0.8 | 4 (3,4) | **SS1-4*** | SS3-a | 1.1 ± 0.2 | 1 (1,1) | **SS3-1** |
|  | SS1-e | 3.5 ± 1.1 | 3 (3, 4.5) |  | SS3-b | 1.7 ± 0.6 | 2 (1,2) | **SS3-2** |
|  | SS2-a | 1.0 ± 0.0 | 1 (1,1) | **SS2-1** | SS3-c | 2.9 ± 0.6 | 3 (3,3) | **SS3-3** |
|  | SS2-b | 2.0 ± 0.8 | 2 (1.5, 2) | **SS2-2** | SS3-d | 3.7 ±0.5 | 4 (3,4) | **SS3-4** |
|  |  |  |  |  | SS3-e | 4.4 ±0.7 | 5 (4,5) | **SS3-5** |
| **Information Sharing** | IS1-a | 1.2 ± 0.5 | 1 (1,1) | **IS1-1** | IS2-a | 1.1 ± 0.5 | 1 (1,1) | **IS2-1** |
|  | IS1-b | 1.5 ± 0.7 | 1 (1,2) | **IS1-2** | IS2-b | 2.2 ± 0.7 | 2 (2,3) | **IS2-3^#^** |
|  | IS1-c | 3.5 ± 0.9 | 3.5 (3,4) | **IS1-3** | IS2-c | 3.7 ± 0.6 | 4 (3,4) | **IS2-4** |
|  | IS1-d | 3.6 ± 0.9 | 4 (3,4) | **IS1-4** |  |  |  |  |
|  | IS1-e | 4.3 ± 1.0 | 5 (4,5) | **IS1-5** |  |  |  |  |
| **Wrapping Up** | WU1 | -- | -- | **WU1-1,2,3,4,5** | WU2-c | 3.7 ± 0.6 | 4 (3,4) | **WU2-4** |
|  | WU2-a | 1.1 ± 0.5 | 1 (1,1) | **WU2 – 1** | WU3 | - | - | **WU3-1,2,3,4** |
|  | WU2-b | 2.2 ± 0.7 | 2 (2,3) | **WU2-3^#^** |  |  |  |  |
| **Emotions and Values** | EV1-a | 1.1 ± 0.2 | 1 (1,1) | **EV1-1** | EV3-a | 1.1 ± 0.4 | 1 (1,1) | **EV3-1** |
|  | EV1-b | 2.2 ±0.6 | 2 (2,3) | **EV1-2** | EV3-b | 2.3 ± 0.8 | 2 (2,3) | **EV3-2** |
|  | EV1-c | 3.2 ± 0.7 | 3 (3,3.5) | **EV1-3** | EV3-c | 3.4 ± 0.8 | 3 (3,4) | **EV3-3** |
|  | EV1-d | 3.5 ± 0.7 | 4 (3,4) | **EV1-4** | EV3-d | 3.8 ± 0.7 | 4 (3,4) | **EV3-4** |
|  | EV2 | - | - | **EV2 -1,2,3** | EV3-e | 4.5 ± 0.7 | 5 (4,5) | **EV3-5** |
|  | EV2-e | 4.3 ± 0.6 | 4 (4,5) | **EV2-4** |  |  |  |  |
| **Communication** | C1-a | 1.1 ± 0.2 | 1 (1,1) | **Com1-1** | C2-a | 1.6 ± 0.5 | 2 (1,2) | Com2-2 |
|  | C1-b | 1.9 ± 0.4 | 2 (2,2) | **Com1-2** | C2 | - | - | Com2-3 |
|  | C1-c | 2.9 ± 0.6 | 3 (3,3) | **Com1-3** | C2-b | 3.6 ± 0.4 | 4 (3,4) | Com2-4 |
|  | C1-d | 3.8 ± 0.7 | 4 (3.5,4) | **Com1-4*** | C2-c | 4.4 ±0.7 | 5 (4,5) | Com2-5 |
|  | C2 | - | - | Com2-1 | C3 | -- | -- | **Com3-1,2,3,4** |

*Skills combined due to single milestone due to overlapping level

^Skill moved to another subgroup based on expert comments

^#^Skill allowed to span 2 milestone levels

- Skill created from content expert comments

-- Skill created later based on expert observer and participant comments
